# Serum albumin and the short-term mortality in individuals with congestive heart failure in intensive care unit: an analysis of MIMIC

**DOI:** 10.1038/s41598-022-20600-1

**Published:** 2022-09-28

**Authors:** Peng Chao, Xinyue Cui, Shanshan Wang, Lei Zhang, Qingru Ma, Xueqin Zhang

**Affiliations:** 1 Department of Cardiology, People’s Hospital of Xinjiang Uygur Autonomous Region, Xinjiang, China; 2 Department of Nephrology, People’s Hospital of Xinjiang Uygur Autonomous Region, Xinjiang, China; 3 Xinjiang Medical University, Xinjiang, China

**Keywords:** Cardiology, Diseases

## Abstract

Decreased albumin levels are common in congestive heart failure (CHF), but little is known about its role in mortality risk in CHF. This study developed a cohort prediction model based on 7121 individuals with heart failure to evaluate the short-term mortality and prognostic role of albumin in patients with CHF. The cohort was from intensive care unit between 2001 and 2012 in a publicly available clinical database in intensive care called MIMIC III. We used a generalized additive model to determine the nonlinear correlation between serum albumin and 14th day, 28th day and 90th day all-cause mortality in patients with heart failure. The results showed that serum albumin is an independent risk factor for 14th, 28th and 90th day all-cause mortality, and has a linear relationship with all-cause mortality in congestive heart failure. Cox regression analysis using restricted cubic spline with albumin as continuous parameter showed that the decrease of albumin level is directly related to the increase of mortality (14th day mortality: hazard ratio [HR], 0.65 [95% *CI*, 0.58 to 0.73]); 28th day mortality: HR, 0.56 [95% *CI*, 0.51 to 0.63]; 90th day mortality: HR, 0.52 [95% *CI*, 0.47 to 0.57]; *P* for trend < 0.001). The multivariate adjusted association between albumin (as a continuous variable) and all-cause mortality on the 90th days is mixed by ARDS [HR, 0.64, 95% *CI* (0.47–0.87), *P* = 0.005]. The all-cause mortality on the 90th day predicted better clinical results with the all-cause mortality on the 14th day.

## Introduction

Heart failure (HF), a major epidemic and public health burden, associated with considerable morbidity and mortality^1^. In the United States, approximately 10–51% of hospitalized patients with HF were reported to be admitted to the ICU, with a mortality rate of 10.6%^2–4^. Inadequate cardiac output may result in congestive heart failure (CHF), which is a pathological condition that impairs tissue and organ perfusion and metabolic activity. Because of the higher in-hospital mortality in ICU-treated HF patients, accurate prognosis prediction and close follow-up may be more beneficial for ICU-treated HF patients.

Hypoalbuminemia is highly prevalent in patients with systolic heart failure, which occurs in about one-third of patients^5^. Hypoalbuminemia in HF patients may be caused by hemodilution, malnutrition, chronic inflammation, infection, proteinuria and other mechanisms^5^. Meanwhile, hypoalbuminemia may leads to the decrease of colloid osmotic pressure and affect the degree of pulmonary congestion and the symptoms of heart failure^6^. In addition, hypoalbuminemia is known to be associated with poor prognosis for end-stage renal disease, infection and cancer, as well as in the elderly^7^. Therefore, Serum albumin levels may be a predictor of mortality in HF patients in the ICU. Tamara B et al.^8,9^ found that comparison to patients without hypoalbuminemia, the risk of death in patients suffering late systolic HF with hypoalbuminemia increased by more than 2 times. Israel G et al.^10^ found that the decrease of albumin was an independent predictor of mortality. The upward trend of serum albumin in patients with acute heart failure is related to a good long-term prognosis^11^. Albumin is a protective factor for in-hospital or long-term mortality of patients with heart failure. However, to our best knowledge, there is an unclear conclusion about the short-term mortality of systolic heart failure.

Although a variety of in-hospital mortality prediction models are currently available, none of these methods have satisfactory accuracy and have not been widely used^12–14^. In addition, there are limited data on predictive models for the association of serum albumin levels with mortality in ICU-treated HF patients. Therefore, we used data from the MIMIC III database to develop a predictive model of serum albumin levels and short-term mortality from systolic heart failure in the intensive care unit (ICU).

## Methods

### Data base

Our research was based on an open intensive care clinical database named Multi-parameter Intelligent Monitoring III^15^. The database included more than 40,000 intensive care patients admitted to Beth Israel Deaconess Medical Center (Boston, Massachusetts, USA) from 2001 to 2012. In order to apply for access to the database, we completed the online course of the National Institutes of Health in the United States and passed the test of human protection research participants (No.6182750). The project was approved by Beth Israel Deaconess Medical Center and the Institutional Review Committee of Massachusetts Institute of Technology (Cambridge, Massachusetts). In order to protect the privacy of patients, the data was de-identified. Therefore, informed consent was abandoned.

### Study population

**Figure 1:**
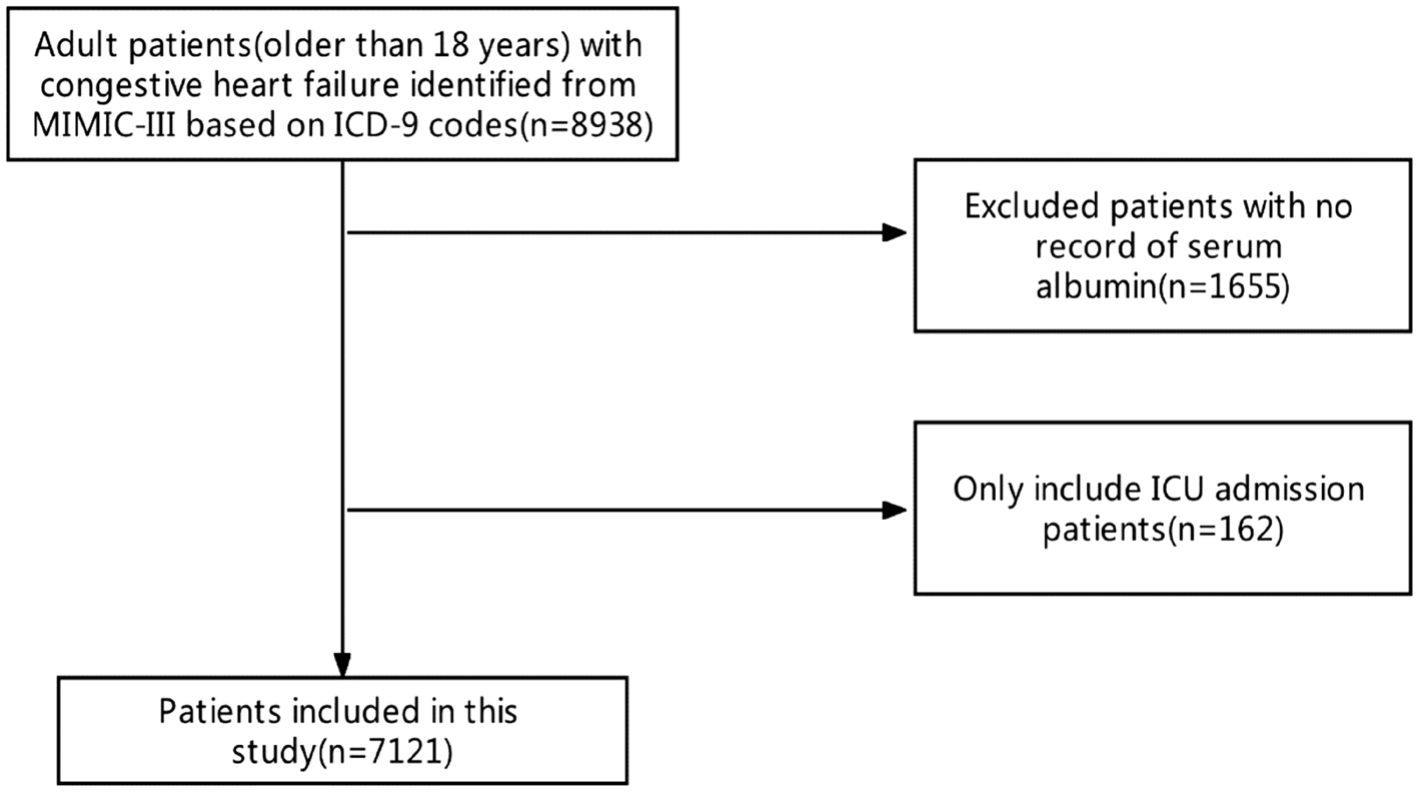
Flow diagram of patient included in the study.

Using ICD-9 disease codes, we analyzed the database for adult CHF patients at the time of first ICU admission. Patients with CHF diagnostic criteria of the European Society of Cardiology (ESC) will be included^16^. This standard is also based on the typical symptoms, signs and structural or functional abnormalities of the heart during echocardiography. Exclusion criteria include patients who are younger than 18 years old, stay in ICU for less than 24 h or have no corresponding laboratory test results or data needed for research (Fig. 1). We extracted clinical variables, including demographic characteristics, ICD-9 code, physiological indicators, drugs and laboratory tests. Because the birthdate of patients over the age of in the database was accurately moved to 300 years ago to cover their age, it was modified (age −300 + 89) before the analysis. All scores were based on the lowest difference of relevant data within 24 h in ICU. Patients who had been admitted to intensive care unit for many times only include their initial admission results.

### Exposure and outcomes

The main exposure was mean serum albumin, baseline albumin, maximum albumin and minimum albumin value, in which 7121 participants were measured. As the four albumin levels have basically the same trend on short-term death (data have been presented in supplementary data), the average albumin was selected to represent the correlation between albumin and short-term death in this study (Fig. S1-6,Table S1-3). The mortality on the 14th, 28th and 90th day following admission to ICU was selected as the primary endpoint, and the mortality of ICU was determined only by the first admission to ICU.

### Covariates

Use PostgreSQL (version 9.6) Structured query language for extracting data from MIMIC III. Demography, vital signs, laboratory tests, complications, scoring system and other variables collected 24 h before admission to intensive care unit were taken from MIMIC III. Co-diseases include coronary artery disease, hypertension, acute respiratory distress syndrome, diabetes, cigarette, alcohol abuse, chronic obstructive pulmonary disease, BNP, LVEF, diuretic, ACEI/ARB/ARNI and cardiac glycoside.

### Statistical analysis

Descriptive statistics were summarized as the average and standard deviation or median of continuous variables (quartile distance), and frequency distribution was expressed as a percentage of classified variables. *Chi-square* test, *one-way ANOVA* and *Kruskar-Wallis H* test were used to determine the significant differences among groups. Generalized additive model (GAM) was used to determine the non-linear relationship between serum albumin and all-cause mortality. Cox proportional risk model was used to determine the relationship between albumin level and mortality of congestive heart failure at the 14th, 28th and 90th day. These results were expressed by the risk ratio of *95% CI*. Multiple imputation was used to deal with the missing data.

The timing of events was analyzed to check the outcome risk of albumin quartile (the lowest quartile in the reference group). In Cox model, the statistical interaction between serum and ARDS was tested by multiplying the interaction term. Less than 10% of the covariant data was lost, so we didn't use interpolation technology. The proportional risk hypothesis was evaluated in all models by Kolmogorov-type supremum test. The main follow-up of the analysis focused on the all-cause mortality results of the 14th, 28th and 90th day in the intensive care unit. According to ARDS subgroup analysis, Wald statistics of cross product terms of trend variables and subgroup members were used to test the heterogeneity among subgroups. All probability values were bilateral, and values less than 0.05 were considered statistically significant. R (http://www.R-project.org) was used for all statistical analysis.

## Result

### Baseline characteristics

A total of 7121 patients participated in this study. Table 1 lists the baseline characteristics of the study population according to average albumin level. The patients were characterized by more advanced in age, with more males than females. Heart rate, respiratory rate, platelet, creatinine, leukocyte, aspartate transaminase (AST), hypertension and acute respiratory distress syndrome (ARDS) in patients with high average serum albumin were lower than those in patients with low average albumin, while systolic blood pressure, diastolic blood pressure, mean arterial pressure, hemoglobin, diabetes and coronary heart disease (CHD) were higher than those in patients with low albumin (as shown in Table 1).

**Table 1:** Characteristics of the participants.

Characteristics	Q1(n = 1780) (≤ 0.94)	Q2(n = 1780) (0.94–1.29)	Q3(n = 1780) (1.29–1.90)	Q4(n = 1781) (1.90–15.53)	*P*
Age(years)	93.79 ± 69.09	93.12 ± 68.32	95.66 ± 71.49	87.07 ± 62.87	< 0.001
Heart Rate (bpm)	88.21 ± 16.55	85.07 ± 15.91	83.53 ± 15.20	83.75 ± 14.52	< 0.001
SBP (mmHg)	113.57 ± 16.90	116.54 ± 17.94	117.82 ± 17.01	117.78 ± 16.71	< 0.001
DBP (mmHg)	55.36 ± 9.64	57.44 ± 10.91	58.18 ± 10.55	58.98 ± 10.60	< 0.001
MAP (mmHg)	73.52 ± 10.57	75.47 ± 11.54	76.05 ± 10.56	76.49 ± 10.65	< 0.001
Respiratory Rate (bpm)	19.88 ± 4.52	19.84 ± 4.26	19.58 ± 3.91	19.06 ± 3.75	< 0.001
Temperature (℃)	36.76 ± 0.72	36.76 ± 0.68	36.74 ± 0.61	36.79 ± 0.58	0.13
SPO2 (mmHg)	96.82 ± 3.59	96.83 ± 3.25	96.91 ± 2.25	97.03 ± 2.05	0.14
Platelet(10^9^/L)	245.73 ± 114.77	238.93 ± 103.91	230.13 ± 92.44	223.35 ± 82.10	< 0.001
Blood glucose (mg/dL)	134.09 ± 34.78	133.60 ± 31.91	134.71 ± 36.82	133.11 ± 33.30	0.544
Hemoglobin (g/dL)	10.07 ± 1.15	10.41 ± 1.33	10.64 ± 1.39	11.00 ± 1.49	< 0.001
Cr(mg/dL)	1.71 ± 1.36	1.71 ± 1.36	1.67 ± 1.38	1.57 ± 1.36	< 0.001
WBC (10^9^/L)	12.63 ± 8.50	10.98 ± 5.08	10.37 ± 5.39	10.08 ± 4.85	< 0.001
BNP (pg/dL)	11,041.19 ± 15,004.42	7470.33 ± 9505.22	6894.30 ± 10,285.01	8756.05 ± 15,036.94	0.108
LVEF(%)	34.13 ± 12.26	38.88 ± 15.23	41.32 ± 13,23	45.33 ± 1`8.77	< 0.001
AST (umol/L)	124.82 ± 586.94	109.74 ± 532.73	96.25 ± 317.29	88.44 ± 351.15	0.003
Men(n, %)	910 (51.12%)	966 (54.27%)	958 (53.82%)	1002 (56.26%)	0.022
Hypertensive(n, %)	98 (34.27%)	92 (31.72%)	73 (25.26%)	82 (28.47%)	< 0.001
Diabetes(n, %)	611 (34.33%)	672 (37.75%)	727 (40.84%)	661 (37.11%)	< 0.001
CHD(n, %)	527 (29.61%)	680 (38.20%)	855 (48.03%)	874 (49.07%)	< 0.001
COPD(n, %)	74 (4.16%)	71 (3.99%)	87 (4.89%)	70 (3.93%)	0.464
ARDS(n, %)	203 (11.40%)	120 (6.74%)	72 (4.04%)	77 (4.32%)	< 0.001
**Cigarette**					0.525
1	135 (40.91%)	161 (35.46%)	196 (40.00%)	192 (36.29%)	
2	46 (13.94%)	73 (16.08%)	71 (14.49%)	87 (16.45%)	
3	14 (4.24%)	25 (5.51%)	19 (3.88%)	16 (3.02%)	
4	135 (40.91%)	195 (42.95%)	204 (41.63%)	234 (44.23%)	
Alcohol abuse (%)	22 (100.00%)	18 (100.00%)	23 (100.00%)	27 (100.00%)	0.61
diuretic	1386(77.87%)	1405(78.93%)	1512(84.94%)	1521(85.40%)	< 0.001
ACEI/ARB/ARNI	1392(78.20%)	1404(78.88%)	1508(84.72%)	1534(86.13%)	< 0.001
cardiac glycoside	616(34.61%)	574(32.25%)	504(28.31%)	481(27.01%)	< 0.001
Potassium(mmol/l)	3.82 ± 1.38	4.16 ± 2.58	3.68 ± 1.44	4.58 ± 1.23	0.733
Sodium(mmol/l)	138.73 ± 81.32	142.73 ± 88.79	144.58 ± 91.23	148.82 ± 93.23	< 0.001

Note: Continous variables are expressed in terms of mean ± SD. The weighted linear regression model was used to determine the *P* value. Categorical variables are presented in terms of %. The weighted chi-square test was used to determine *P* value. SBP, systolic blood pressure; DBP, systolic blood pressure; MAP, mean arterial pressure; SPO2, pulse oxygen saturation; BUN, blood urea nitrogen; Cr, creatinine; WBC, white blood cell; BNP, B-type natriuretic peptide; AST, aspartate transaminase; CHD, coronary heart disease; ARDS, acute respiratory distress syndrome; COPD, chronic obstructive pulmonary disease, BNP: B-type natriuretic peptide, LVEF: left ventricular ejection fraction, diuretic, ACEI/ARB/ARNI, cardiac glycoside.

### Survival analysis

**Figure 2:**
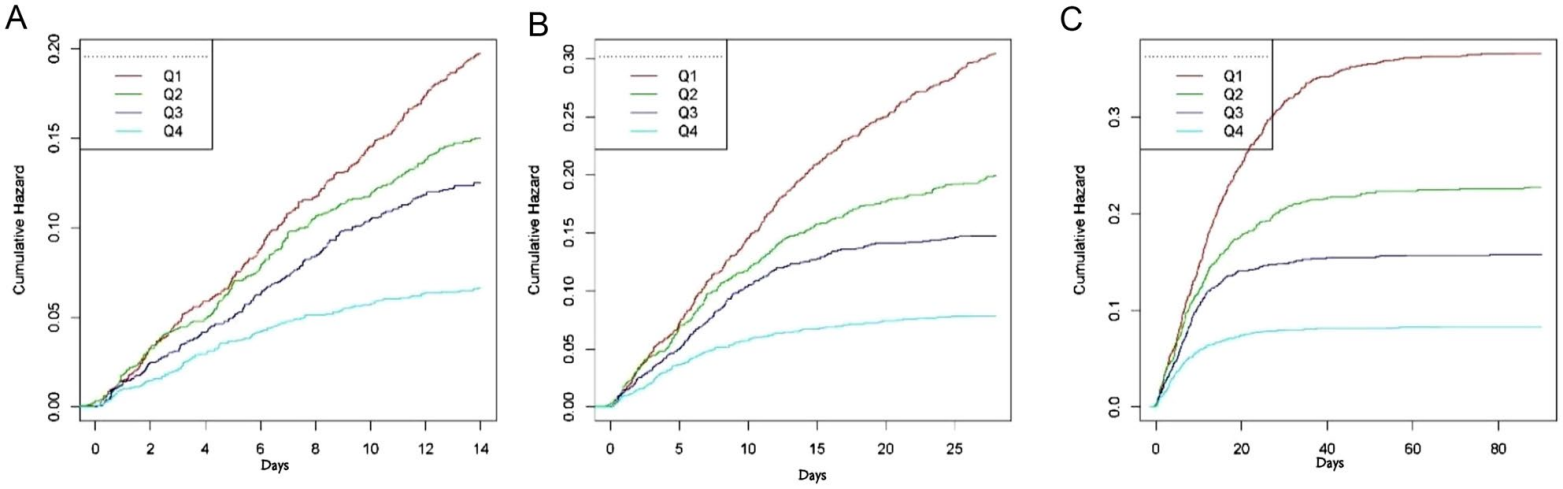
Kaplan–Meier chart of probability of cumulative hazard among the different average albumin levels. (**A**) Kaplan–Meier chart of probability of 14th day survival among the different albumin levels. (**B**) Kaplan–Meier chart of probability of 28th day survival among the different albumin levels. (**A**) Kaplan–Meier chart of probability of 90th day survival among the different albumin levels.

All-cause mortality was 12.5%, 16.8% and 18.4% on the 14th, 28th and 90th day after admission, respectively (Table S4). As shown in Kaplan–Meier survival curves in Fig. 2 and Table 2, unadjusted and multivariate adjusted risk ratios and *95% CI* calculated according to average albumin and quartiles as continuous variables. The results showed that the average albumin as a continuous variable was related to all-cause mortality at the 14th day, 28th day and 90th day(HR 0.58, 95% CI0.53–0.64, *P* < 0.0001; HR0.49, 95% CI 0.45–0.54, *P* < 0.0001; HR0.46 ,95% CI 0.42–0.50, *P* < 0.0001). For every standard deviation increase of albumin, the all-cause mortality decreased by 42%, 51% and 54% on 14th, 28th and 90th days. After adjustment of heart rate, SBP, mean arterial pressure, diastolic pressure, breathe rate, temperature, SPO2, platelet, potassium, sodium, creatinine, hemoglobin, WBC, AST, BNP, LVEF, diuretic, ACEI/ARB/ARNI and cardiac glycoside, the correlation between average albumin and all-cause mortality was weakened as a continuous variable and quartile(continuous variable:HR 0.64, 95% CI 0.58–0.71, *P* < 0.0001; HR 0.56 95% CI 0.51–0.61, *P* < 0.0001;HR 0.52,95% CI 0.48–0.57, *P* < 0.0001). For every standard deviation increase of albumin, the all-cause mortality decreased by 36%, 44% and 48% on 14th, 28th and 90th days. ARDS significantly confuses the relationship between average albumin (as a continuous variable) and all-cause mortality. The results of *P* value for trend showed that all-cause mortality decreased with the increase of average albumin on the 14th, 28th and 90th day.

**Table 2:** Average albumin and the risk of all-cause mortality by Cox proportional hazards.

	HR, 95%CI, P								
	Model 1			Model 2			Model 3		
	14th day	28th day	90th day	14th day	28th day	90th day	14th day	28th day	90th day
Continuous	0.58 (0.53, 0.64) < 0.0001	0.49 (0.45, 0.54) < 0.0001	0.46 (0.42, 0.50) < 0.0001	0.64 (0.58, 0.71) < 0.0001	0.56 (0.51, 0.61) < 0.0001	0.52 (0.48, 0.57) < 0.0001	0.63 (0.46, 0.67) < 0.0001	0.59 (0.59, 0.68) < 0.0001	0.52 (0.43, 0.61) < 0.0001
**Categorical**									
Q1	1.0	1.0	1.0	1.0	1.0	1.0	1.0	1.0	1.0
Q2	0.77 (0.65, 0.91) 0.0018	0.67 (0.58, 0.77) < 0.0001	0.64 (0.56, 0.73) < 0.0001	0.83 (0.70, 0.98) 0.0280	0.74 (0.64, 0.86) < 0.0001	0.72 (0.63, 0.82) < 0.0001	0.88 (0.69, 0.99) 0.0093	0.74 (0.63, 0.97) < 0.0001	0.71 (0.58, 0.93) < 0.0001
Q3	0.64 (0.54, 0.76) < 0.0001	0.50 (0.43, 0.58) < 0.0001	0.45 (0.39, 0.52) < 0.0001	0.71 (0.60, 0.85) 0.0002	0.58 (0.49, 0.68) < 0.0001	0.53 (0.46, 0.62) < 0.0001	0.71 (0.62, 0.89) 0.0006	0.63 (0.58, 0.77) < 0.0001	0.59 (0.49, 0.66) < 0.0001
Q4	0.34 (0.28, 0.42) < 0.0001	0.27 (0.22, 0.32) < 0.0001	0.24 (0.20, 0.28) < 0.0001	0.42 (0.33, 0.52) < 0.0001	0.34 (0.28, 0.41) < 0.0001	0.31 (0.25, 0.37) < 0.0001	0.59 (0.58, 0.69) < 0.0001	0.41 (0.35, 0.59) < 0.0001	0.39 (0.29, 0.48) < 0.0001
*P* for trend	< 0.0001	< 0.0001	< 0.0001	< 0.0001	< 0.0001	< 0.0001	< 0.0001	< 0.0001	< 0.0001
Change per quartile	0.70 (0.66, 0.75) < 0.0001	0.63 (0.60, 0.67) < 0.0001	0.60 (0.57, 0.64) < 0.0001	0.75 (0.70, 0.81) < 0.0001	0.69 (0.65, 0.73) < 0.0001	0.66 (0.63, 0.70) < 0.0001	0.83 (0.79, 0.91) < 0.0001	0.77 (0.71, 0.85) < 0.0001	0.71 (0.69, 0.88) < 0.0001

Model 1: adjust for none.

Model 2: adjust for gender; coronary; age; hypertension; diabetes; ARDS; COPD; renal failure.

Model3: adjust for heart rate; SBP; mean arterial pressure; diastolic pressure; respire rate; temperature; SPO2; platelet; potassium; sodium; creatinine; hemoglobin; WBC; AST, BNP, LVEF, diuretic, ACEI/ARB/ARNI, cardiac glycoside.

**Figure 3:**
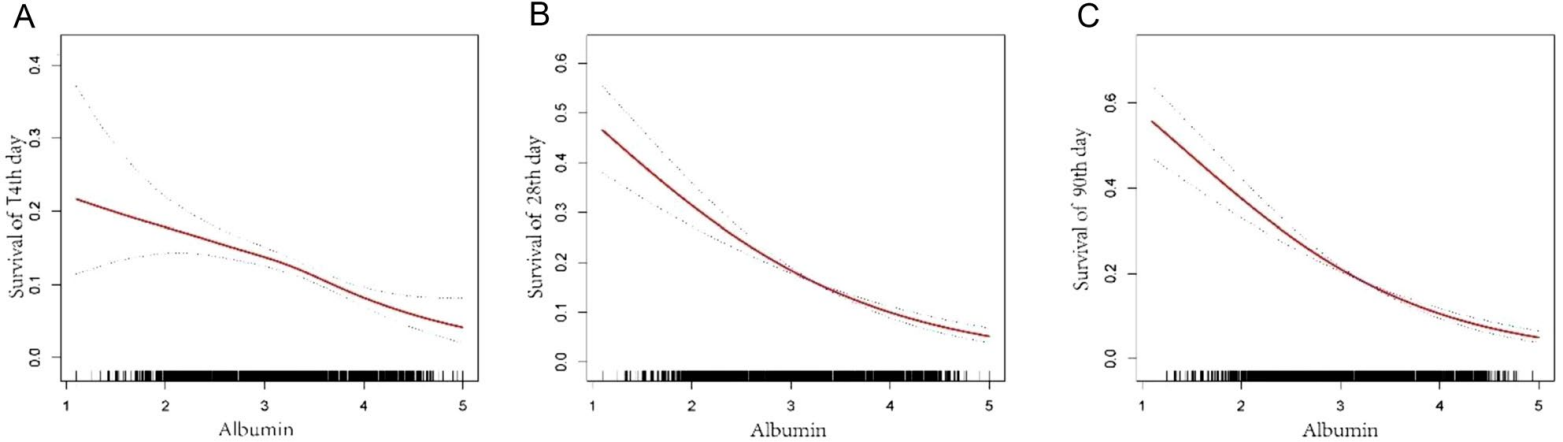
Restricted cubic spline curve showing the adjusted hazard ratios for all-cause mortality as a function of average albumin concentration. Albumin as a continuous variable, model adjusted for heart rate; SBP; mean arterial pressure; diastolic pressure; respire rate; temperature; SPO2; platelet; potassium; sodium; creatinine; hemoglobin; WBC; AST, BNP, LVEF, diuretic, ACEI/ARB/ARNI, cardiac glycoside. At the figure, the frequency distribution of albumin is shown.

After adjusting the covariates of heart rate, SBP, mean arterial pressure, diastolic pressure, respiratory rate, temperature, SPO2, platelet, potassium, sodium, creatinine, hemoglobin, WBC, AST, BNP, LVEF, diuretic, ACEI/ARB/ARNI and cardiac glycoside, we observed that there was a linear relationship between average albumin level and all-cause mortality on 14th, 28th and 90th day (HR 0.63 95%CI 0.46–0.67, *P* < 0.0001; HR 0.59 95% CI 0.58–0.68, *P* < 0.0001; HR 0.52 95% CI 0.43–0.61, *P* < 0.0001) (Fig. 3). For every quartile standard deviation increase of average serum albumin level, all-cause mortality on the 14th, 28th and 90th day decreased by 24%, 31% and 35% compared with Q1(≤ 0.94dg/ml) (Table 2). In addition, we also found that the threshold of 3.56 mg/dl was related to different death risks, that is, the death risk of average albumin below 3.56 mg/dl was lower than that of average albumin above 3.56 mg/dl (Table S5).

The multivariate adjusted association between average albumin (as a continuous variable) and all-cause mortality at the 14th and 28th day is mixed by ARDS(*P* value of 14th day = 0.0003, *P* value of 28th day = 0.0092, *P* value of 90th day = 0.0412) (Table S6). It was found that average albumin, as far as the participants without ARDS were concerned, had a more obvious negative correlation with all-cause mortality on the 14th and 28th day (Figure S7). However, for patients with ARDS, average albumin had no obvious correlation with all-cause mortality on the 14th and 28th day after adjusting the covariates of heart rate, SBP, mean arterial pressure, diastolic pressure, respiratory rate, temperature, SPO2, platelet, potassium, sodium, creatinine, hemoglobin, WBC, AST, BNP, LVEF, diuretic, ACEI/ARB/ARNI and cardiac glycoside (HR 1.04 95% CI 0.80–1.35, *P* = 0.7858; HR 0.77 95% CI 0.55–1.08, *P* = 1344). However, the negative correlation between average albumin and the 90th day mortality was not affected by ARDS (HR 0.64 95% CI 0.47–0.87, *P* = 0.0047) (Table S7).

## Discussion

The three main findings of this study of serum average albumin level in 7121 intensive care unit patients with congestive heart failure were: (1) Higher average albumin level was found to be independently associated with a higher risk of 14th, 28th and 90th day all-cause mortality in CHF patients through the continuous follow-up. Especially when the average albumin level was above 3.56 mg/dl, the risk of death on 14th day was lower. (2) The negative association between albumin (as a continuous variable) and all-cause mortality at the 14th and 28th day was mixed by ARDS. (3) *P* value for trend was used to statistically test this trend, and Change Per Quartile was used to further quantify this trend.

In the case of acute HF, albumin has been proved to have an important influence on survival^17–19^. Although multiple risk grading models and HF prognostic scores have been proposed and validated, the role of SA has not been investigated^12,20^. In a recent meta-analysis, Peng et al.^21^ found that HA was associated with increased mortality in heart failure. In addition, Mahmoud et al.^22^ found that in-hospital mortality in HF was inversely associated with SA. SA showed a decreasing trend and was associated with worse prognosis in acute and chronic HF^23,24^. Jabbour et al.^25^ followed 212 patients with chronic systolic HF for more than 2 years and found that SA reduction from baseline was associated with higher mortality compared with retention of baseline SA. A study by Biegus et al.^23^ showed that a decreasing trend in SA during the first 4 days of hospitalization was associated with increased mortality at 6 months, and the risk was proportional to the degree of albumin reduction. However, few study surveyed the magnitude and direction of the association between the albumin and mortality continually. Our study revealed linear association between serum albumin and all-cause mortality on 14th, 28th, and 90th days in patients with congestive heart failure respectively. In addition, a study has found that serum albumin levels < 3 mg/dL were associated with decreased renal function during treatment in patients with acute HF^26^. We found the risk of all-cause mortality on day 14 was significantly reduced when albumin was increased above 3.56 mg/dl, which may provide a theoretical reference value for clinical treatment.

Low albumin in patients with heart failure is mainly manifested by reduced albumin synthesis and protein loss, which may be caused by hemodilution, chronic inflammatory states, hepatic congestion, malnutrition, cachexia due to volume overload, and proteinuria or intestinal disease. Albumin is associated with numerous detrimental biological processes which are present in HF and pertain to a worse outcome^27^. Low albumin in heart failure promotes and aggravates congestion due to the decrease of intravascular colloidal osmotic pressure^28^, the increases of oxidative stress^29^, inflammation^30^, and susceptibility to infection. The mechanism may be supposed that S-thiolation of albumin is increased in the plasma of HF patients and induced changes in the structure and antioxidant function of human serum albumin, and provide a new paradigm of the proinflammatory effect of S-thiolation HAS^31^. Therefore, low albumin is the sum of many harmful factors in patients with heart failure, which is expected to provide important prognostic information for patients with heart failure.

Our findings suggested that ARDS and albumin interacted in the prediction of results. Previous studies have found that hypoproteinemia (< 5.9 g/dl) and hypoproteinemia level < 2.4 g/dl were considered as signs of increased lung permeability in patients with sepsis and acute respiratory distress syndrome. The lung leakage index would decrease with the rise of serum protein^32^. In-depth analysis of the results showed that the decrease of colloidal osmotic pressure under normal permeability of vascular endothelium would not cause edema. On the contrary, it was the increased permeability caused by endothelial damage that leads to hypoproteinemia^32^. The increase of systemic permeability caused by low albumin can’t be equated with similar pulmonary vascular permeability. Besides, on the day of admission to ICU, serum albumin has nothing to do with the degree of pulmonary degassing described by LUSS in ARDS patients. Serum albumin level < 3.25 g/dL increases the chances of prolonging ICU stay (≥ 10 days), but it cannot predict the mortality rate^33^. This is also consistent with our conclusion, suggesting that ARDS may be the main risk of all-cause death on the 28th day. Therefore, albumin reduction in patients with initial heart failure may be largely influenced by other factors such as inflammation. In our research, we found that there is a special population in the linear relationship between albumin and all-cause mortality, and the special population refers to the patients with heart failure complicated with ARDS. In patients with heart failure complicated with ARDS, the predictive effect of albumin on all-cause mortality is greatly reduced, and the reason may be related to the close relationship among infection, low albumin and ARDS.

An interesting finding in this study is the extent to which albumin level change over time to the recent death of congestive HF. The all-cause mortality on the 90th day predicted better clinical results with the all-cause mortality on the 14th day. A significantly different finding was reported in the case of chronic HF^25,34^. This finding seems to be biologically unreasonable, as the decline of albumin over time is the characteristic of the degradation state of HF patients. However, the condition of patients tends to be stable after staying in ICU for 14 days, and the mortality is obviously decreasing due to the different death time ranges of our follow-up. This means that in acute cases, these changes are related to the severity, sequelae and progress of acute events. In the chronic environment, these changes may reflect the progress of chronic diseases of albumin, the harmful biological process related to low albumin and its significance to prognosis^34^.

Whether high albumin can effectively reduce the all-cause mortality of congestive HF requires a sufficiently powerful placebo-controlled randomized controlled trial, but there are few studies on whether albumin supplementation can reduce the all-cause death of congestive heart failure patients in intensive care unit, most of them are only observational studies. Experiments showed that administration of albumin would not change the length of stay or mortality of patients in intensive care unit^35,36^. It seems unreasonable that exogenous albumin is expensive to treat hypoalbuminemia. But maybe exogenous albumin cannot stay in the body for a long time.

The most important limitation of our study is related to observation design, which makes it impossible to infer the causal relationship between the observed association between serum albumin and all-cause mortality. Even after multivariable adjustment, residual confusion remains an issue. In addition, there is a lack of the stage of heart failure and the mechanism of death not be assessed. Thirdly, low albumin levels are caused by hemodilution, chronic inflammatory states, hepatic congestion, malnutrition, cachexia due to volume overload, and proteinuria or intestinal disease, there is a deficiency in this study because of the limitation of the database. Fourthly, Because of the limitations of the database, the confounding factors, including acute decompensated heart failure, acute pulmonary edema, cardiogenic shock, acute coronary syndrome complicated by heart failure, were not enrolled in the study. Moreover, further studies are needed to take the influence of low albumin on the pharmacokinetic of the drugs into account. Nevertheless, our advantage lies in the discussion of short-term all-cause mortality, and the serum albumin level has no obvious change with time. We also believe that it is the first time that we have continuously followed up all-cause mortality for 14th, 28th and 90th days.

In conclusion, hypoproteinemia in patients with congestive heart failure in intensive care unit has a linear relationship with the 14th, 28th and 90th day mortality rate, and the recommended range exceeds 3.56g/dl. ARDS strongly confuses the association between albumin and the 14th and 28th day all-cause mortality. Meanwhile, With the passage of time, the all-cause mortality on the 90th day predicted better clinical results with the all-cause mortality on the 14th day. We emphasized the importance of albumin in short-term death of ICU patients with heart failure and provided theoretical threshold to provide a theoretical basis for the treatment goal. Sufficient and powerful randomized, placebo-controlled trials are needed in congestive heart failure of intensive care unit to test whether supplementing endogenous albumin may prove to be an effective method to reduce short-term all-cause mortality.

## Supplementary Information

Supplementary Information.

## Data Availability

Additional data are available in additional documents “Supplementary material.docx”.
