# Supplementary material for: Serum albumin and the short-term mortality in individuals with congestive heart failure in intensive care unit: an analysis of MIMIC
Source: Sci Rep. 2022 Sep 28;12:16251. doi: 10.1038/s41598-022-20600-1 (PMC9519563; doi:10.1038/s41598-022-20600-1)
Supplement: Supplementary file 1 — Supplementary Information. [file 41598_2022_20600_MOESM1_ESM.doc]

### Figure legends


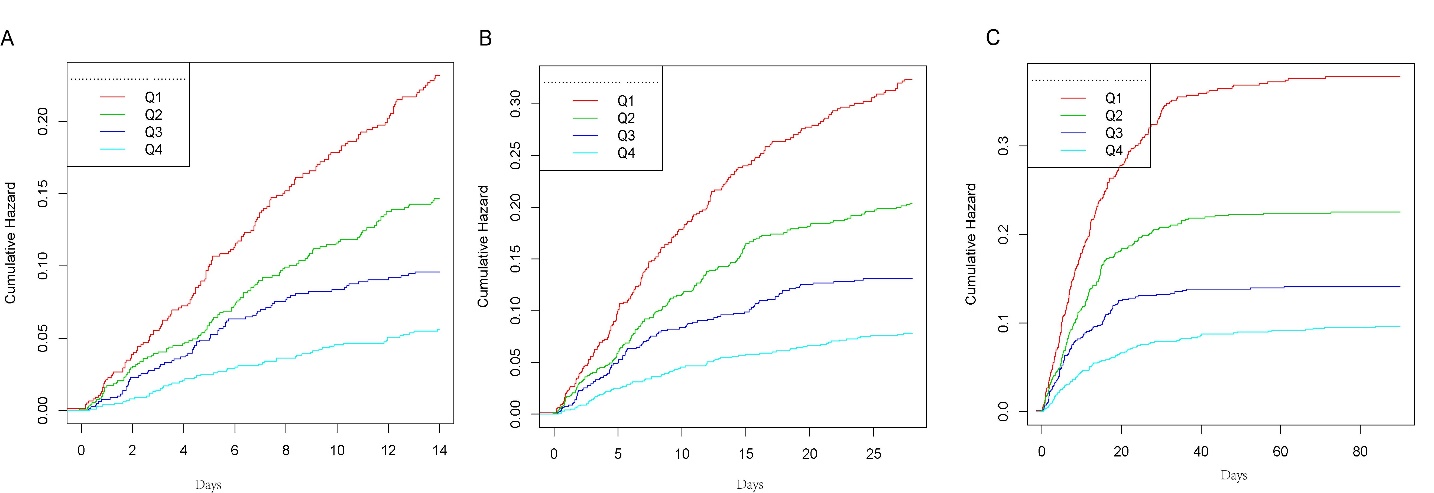


Figure S1. Kaplan-Meier chart of probability of cumulative hazard among the different baseline albumin levels. (A)Kaplan-Meier chart of probability of 14th day survival among the different albumin levels. (B)Kaplan-Meier chart of probability of 28th day survival among the different albumin levels. (A)Kaplan-Meier chart of probability of 90th day survival among the different albumin levels.


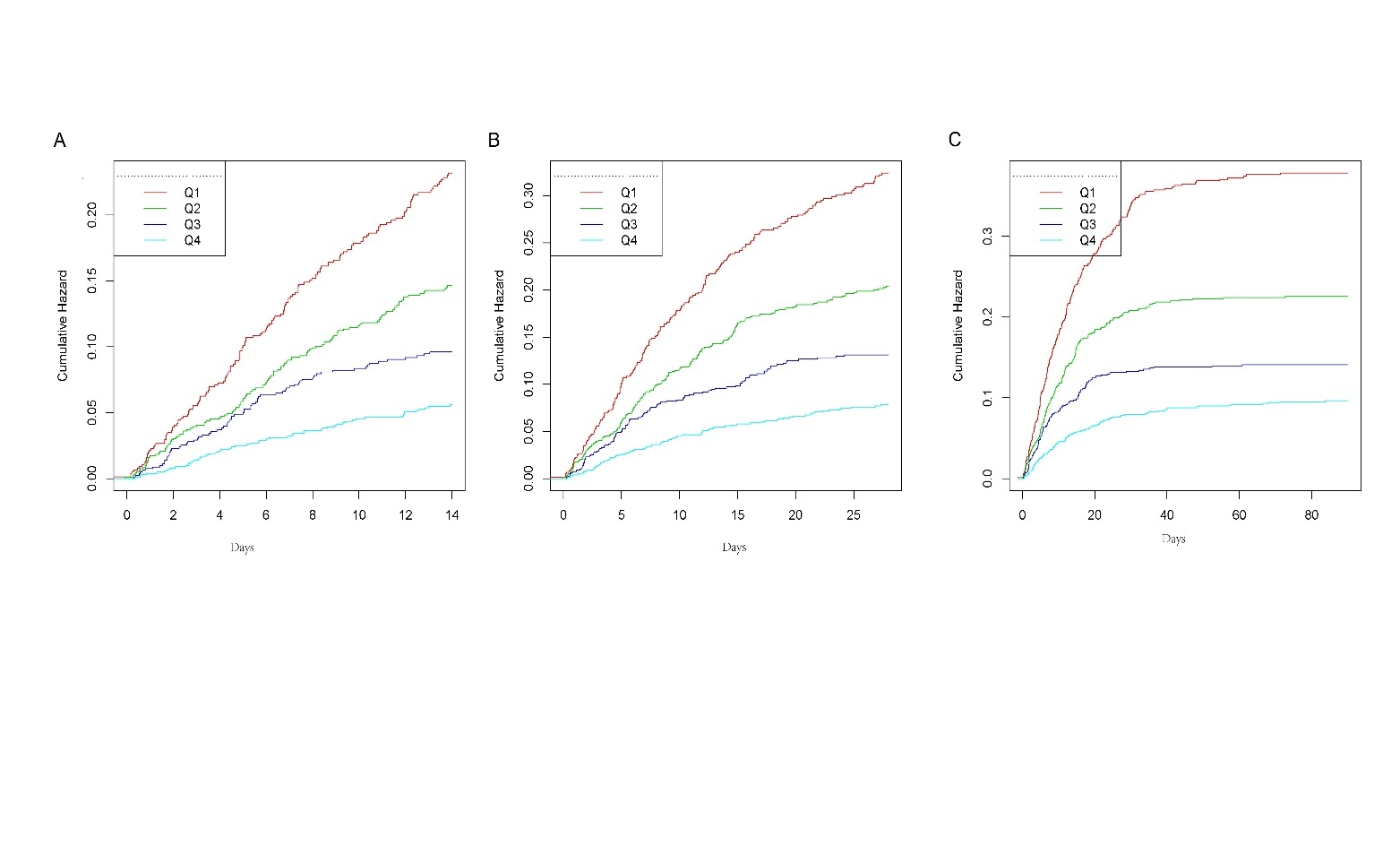


Figure S2. Kaplan-Meier chart of probability of cumulative hazard among the different maximal albumin levels. (A)Kaplan-Meier chart of probability of 14th day survival among the different albumin levels. (B)Kaplan-Meier chart of probability of 28th day survival among the different albumin levels. (A)Kaplan-Meier chart of probability of 90th day survival among the different albumin levels.


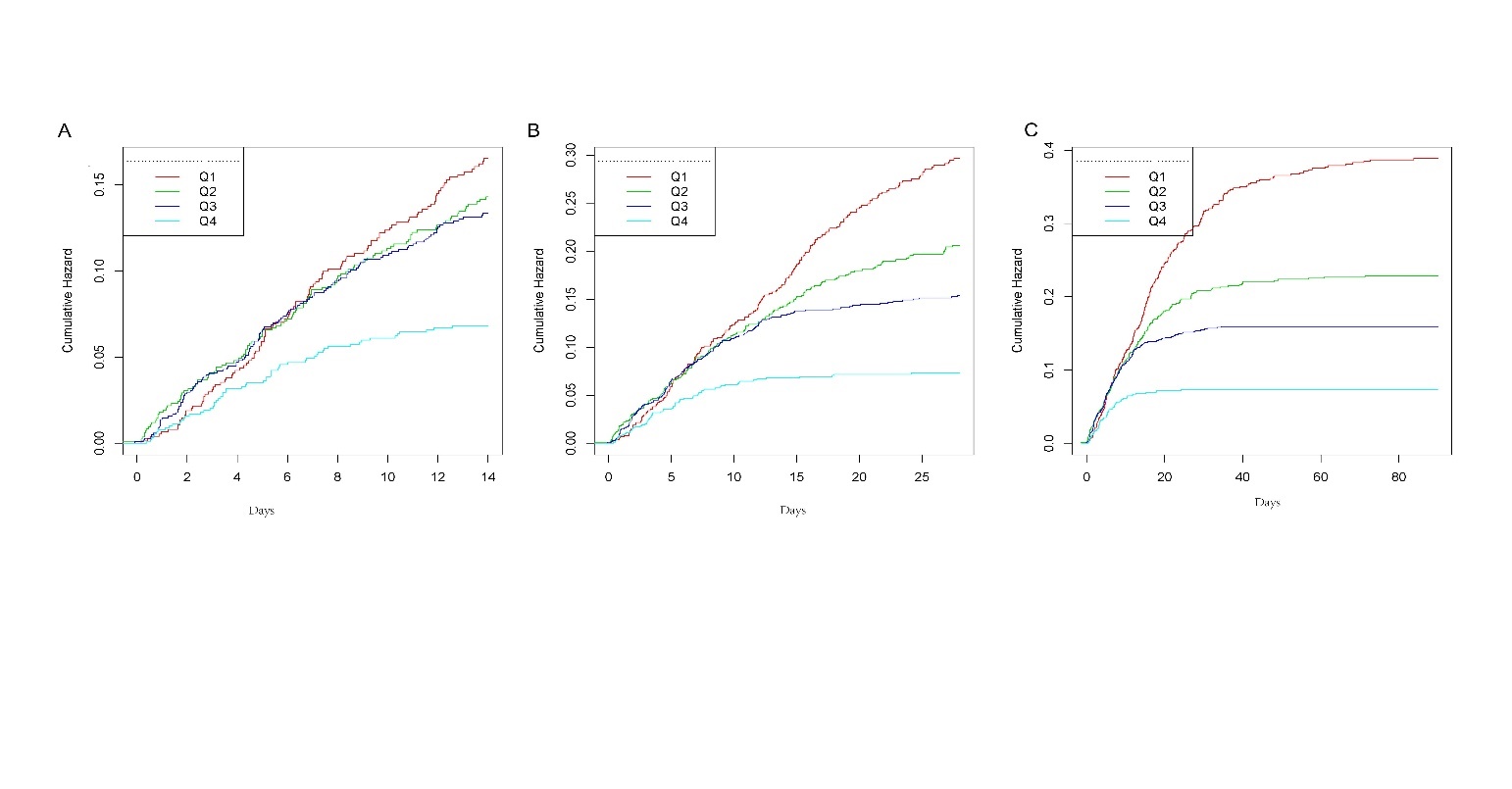


Figure S3. Kaplan-Meier chart of probability of cumulative hazard among the different minimal albumin levels. (A)Kaplan-Meier chart of probability of 14th day survival among the different albumin levels. (B)Kaplan-Meier chart of probability of 28th day survival among the different albumin levels. (A)Kaplan-Meier chart of probability of 90th day survival among the different albumin levels.


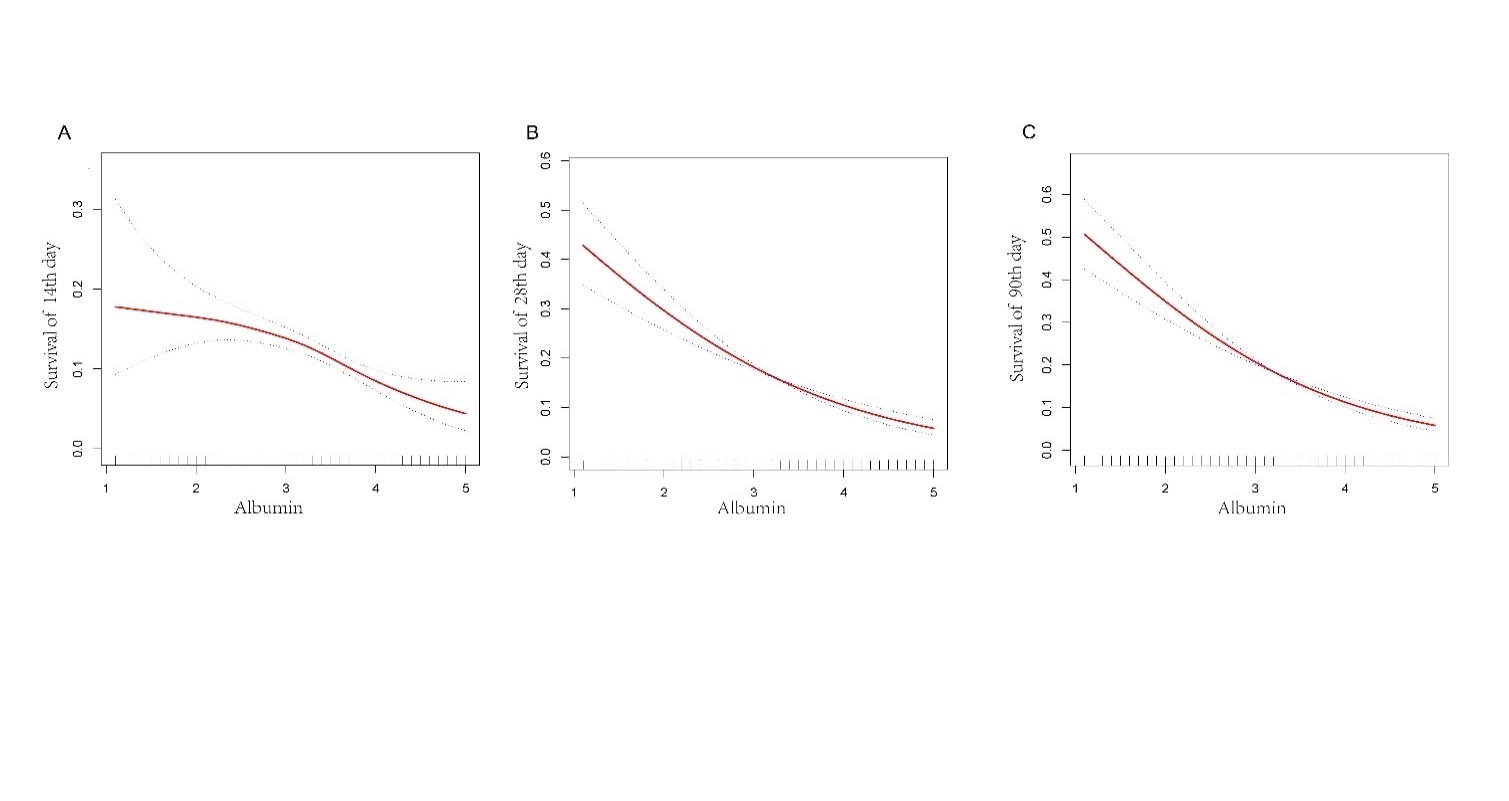


**Figure S4.** Restricted cubic spline curve showing the adjusted hazard ratios for all-cause mortality as a function of baseline albumin concentration. Albumin as a continuous variable, model adjusted for heart rate; SBP; mean arterial pressure; diastolic pressure; respire rate; temperature; SPO2； platelet; potassium; sodium; creatinine; hemoglobin; WBC; AST, BNP, LVEF, diuretic, ACEI/ARB/ARNI, β-blocker. At the figure, the frequency distribution of albumin is shown.


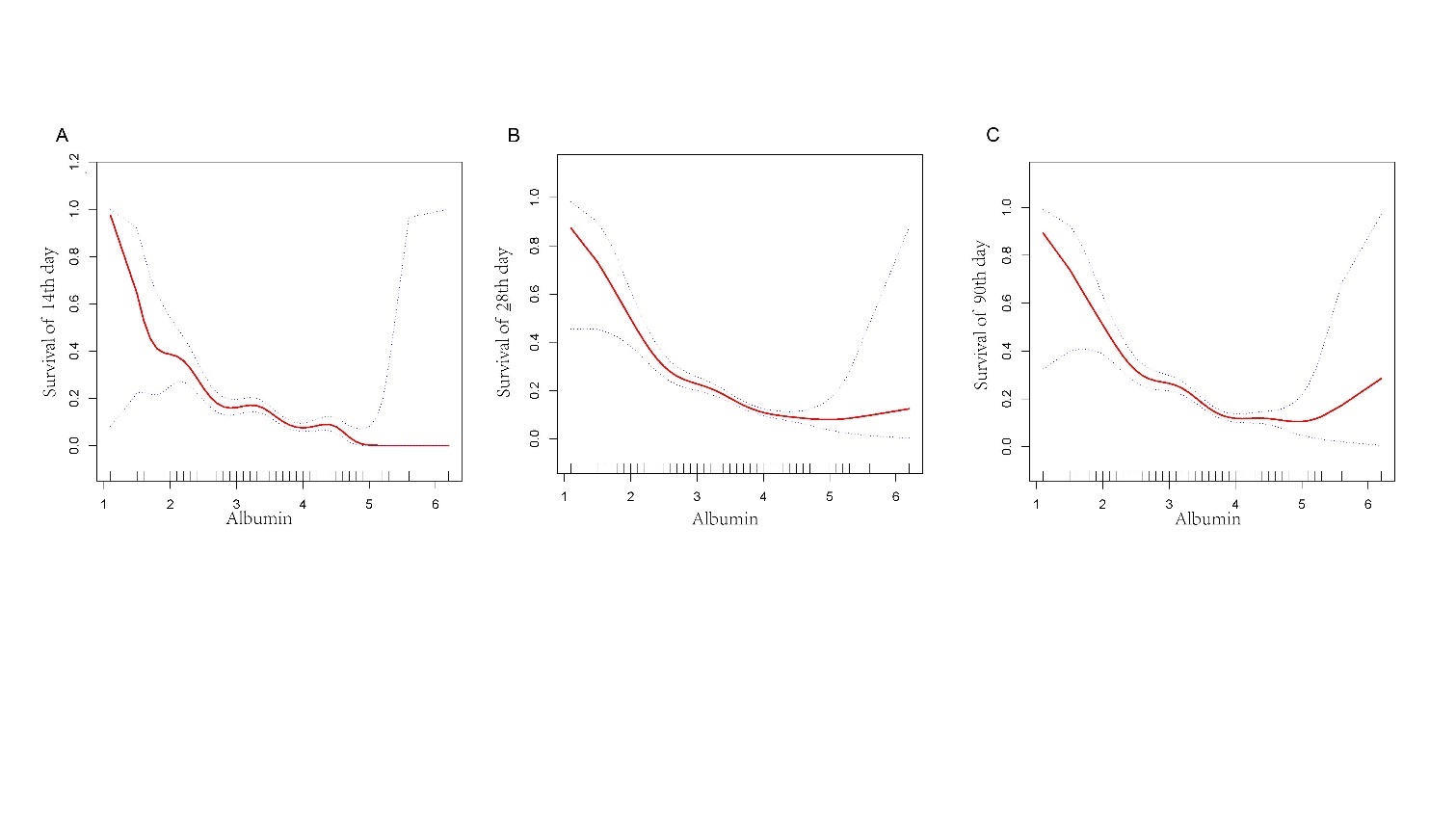


**Figure S5.** Restricted cubic spline curve showing the adjusted hazard ratios for all-cause mortality as a function of maximal albumin concentration. Albumin as a continuous variable, model adjusted for heart rate; SBP; mean arterial pressure; diastolic pressure; respire rate; temperature; SPO2； platelet; potassium; sodium; creatinine; hemoglobin; WBC; AST, BNP, LVEF, diuretic, ACEI/ARB/ARNI, β-blocker. At the figure, the frequency distribution of albumin is shown.


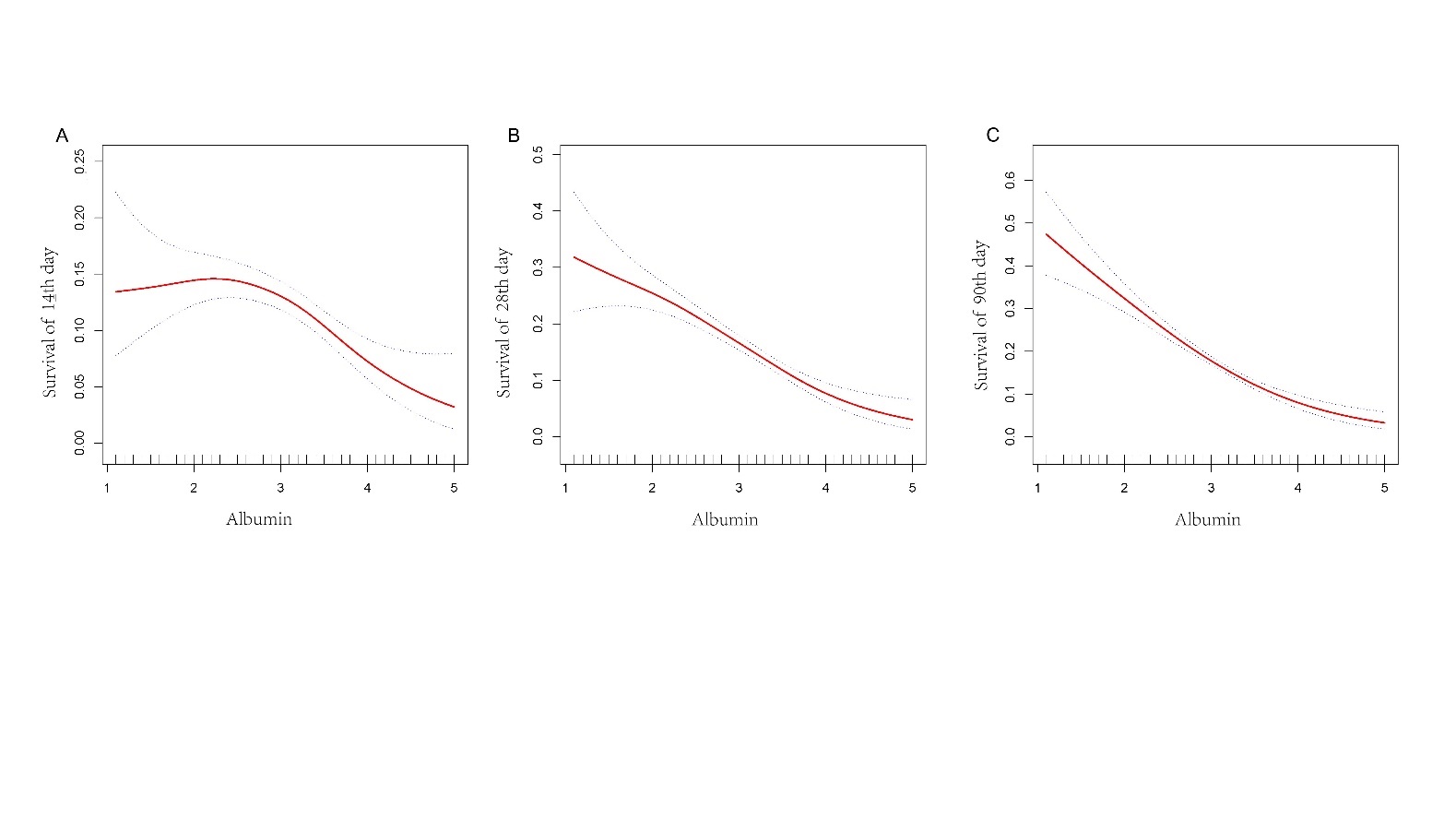


**Figure S6.** Restricted cubic spline curve showing the adjusted hazard ratios for all-cause mortality as a function of minimal albumin concentration. Albumin as a continuous variable, model adjusted for heart rate; SBP; mean arterial pressure; diastolic pressure; respire rate; temperature; SPO2； platelet; potassium; sodium; creatinine; hemoglobin; WBC; AST, BNP, LVEF, diuretic, ACEI/ARB/ARNI, β-blocker. At the figure, the frequency distribution of albumin is shown.

(A) (B) (C)


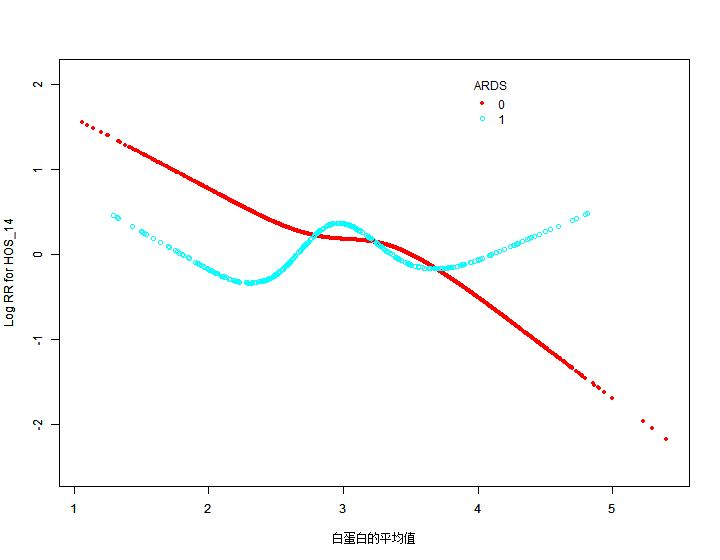

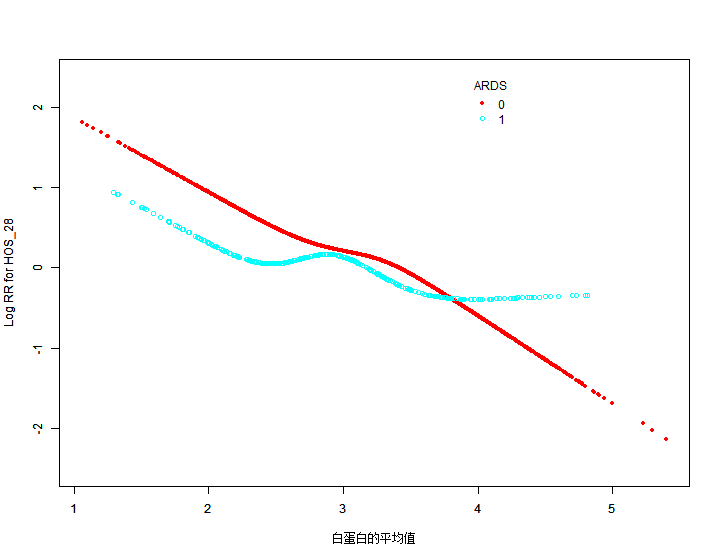

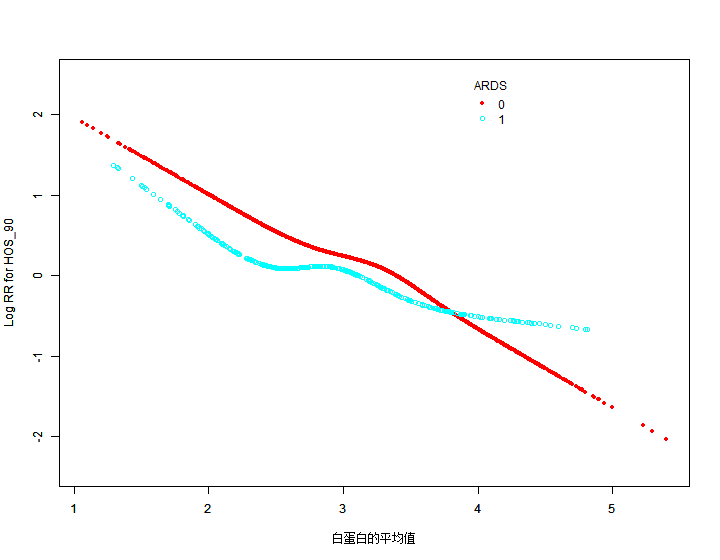


**Figure S7.** Captions Stratified by ARDS. Heart rate, SBP, mean arterial pressure, diastolic pressure, respire rate, temperature, SPO2； platelet, potassium, sodium, creatinine, hemoglobin, WBC, AST were adjusted.

### Tables

Table S1. Baseline albumin and the risk of all-cause mortality by Cox proportional hazards

|  | HR, 95%CI, P | | | | | | | | |
| --- | --- | --- | --- | --- | --- | --- | --- | --- | --- |
|  | Model 1 | | | Model 2 | | | Model 3 | | |
|  | 14th day | 28th day | 90th day | 14th day | 28th day | 90th day | 14th day | 28th day | 90th day |
| Continuous | 0.58 (0.53,  0.64)  <0.0001 | 0.49  (0.45,  0.54)  <0.0001 | 0.46 (0.42,  0.50)  <0.0001 | 0.64 (0.58,  0.71)  <0.0001 | 0.56 (0.51,  0.61)  <0.0001 | 0.52 (0.48,  0.57)  <0.0001 | 0.57 (0.32,  0.78)  <0.0001 | 0.50 (0.29,  0.70)  <0.0001 | 0.44 (0.22,  0.79)  <0.0001 |
| Categorical |  |  |  |  |  |  |  |  |  |
| Q1 | 1.0 | 1.0 | 1.0 | 1.0 | 1.0 | 1.0 | 1.0 | 1.0 | 1.0 |
| Q2 | 0.77 (0.65, 0.91) 0.0018 | 0.67  (0.58,  0.77) <0.0001 | 0.64 (0.56, 0.73) <0.0001 | 0.83 (0.70, 0.98) 0.0280 | 0.74 (0.64, 0.86) <0.0001 | 0.72 (0.63, 0.82) <0.0001 | 0.93 (0.77, 0.99) 0.014 | 0.85 (0.69, 0.98) <0.0001 | 0.77 (0.51, 0.97) <0.0001 |
| Q3 | 0.64 (0.54, 0.76) <0.0001 | 0.50  (0.43,  0.58) <0.0001 | 0.45 (0.39, 0.52) <0.0001 | 0.71 (0.60, 0.85) 0.0002 | 0.58 (0.49, 0.68) <0.0001 | 0.53 (0.46, 0.62) <0.0001 | 0.84 (0.60, 0.89) 0.0006 | 0.76 (0.61, 0.81) <0.0001 | 0.71 (0.64, 0.77) <0.0001 |
| Q4 | 0.34 (0.28, 0.42) <0.0001 | 0.27  (0.22, 0.32) <0.0001 | 0.24 (0.20, 0.28) <0.0001 | 0.42 (0.33, 0.52) <0.0001 | 0.34 (0.28, 0.41) <0.0001 | 0.31 (0.25, 0.37) <0.0001 | 0.71 (0.52, 0.77) <0.0001 | 0.63 (0.44, 0.83) <0.0001 | 0.61 (0.41, 0.77) <0.0001 |
| P for trend | <0.0001 | <0.0001 | <0.0001 | <0.0001 | <0.0001 | <0.0001 | <0.0001 | <0.0001 | <0.0001 |
| Change per quartile | 0.70 (0.66, 0.75)  <0.0001 | 0.63  (0.60,  0.67)  <0.0001 | 0.60 (0.57, 0.64) <0.0001 | 0.75 (0.70, 0.81) <0.0001 | 0.69 (0.65,  0.73)  <0.0001 | 0.66 (0.63,  0.70)  <0.0001 | 0.89 (0.81, 0.91) <0.0001 | 0.80  (0.76,  0.88)  <0.0001 | 0.77 (0.70,  0.86)  <0.0001 |

Model 1: adjust for none.

Model 2: adjust for gender; coronary; age; hypertension; diabetes; ARDS; COPD; renal failure.

Model3: adjust for heart rate; SBP; mean arterial pressure; diastolic pressure; respire rate; temperature; SPO2； platelet; potassium; sodium; creatinine; hemoglobin; WBC; AST, BNP, LVEF, diuretic, ACEI/ARB/ARNI, cardiac glycoside.

Table S2. maximum albumin and the risk of all-cause mortality by Cox proportional hazards

|  | HR, 95%CI, P | | | | | | | | |
| --- | --- | --- | --- | --- | --- | --- | --- | --- | --- |
|  | Model 1 | | | Model 2 | | | Model 3 | | |
|  | 14th day | 28th day | 90th day | 14th day | 28th day | 90th day | 14th day | 28th day | 90th day |
| Continuous | 0.58 (0.53,  0.64)  <0.0001 | 0.49  (0.45,  0.54)  <0.0001 | 0.46 (0.42,  0.50)  <0.0001 | 0.64 (0.58,  0.71)  <0.0001 | 0.56 (0.51,  0.61)  <0.0001 | 0.52 (0.48,  0.57)  <0.0001 | 0.87  (0.67,  0.91)  0.03 | 0.79 (0.64,  0.93)  0.01 | 0.77 (0.61,  0.98)  <0.0001 |
| Categorical |  |  |  |  |  |  |  |  |  |
| Q1 | 1.0 | 1.0 | 1.0 | 1.0 | 1.0 | 1.0 | 1.0 | 1.0 | 1.0 |
| Q2 | 0.77 (0.65, 0.91) 0.0018 | 0.67  (0.58,  0.77) <0.0001 | 0.64 (0.56, 0.73) <0.0001 | 0.83 (0.70, 0.98) 0.0280 | 0.74 (0.64, 0.86) <0.0001 | 0.72 (0.63, 0.82) <0.0001 | 0.82 (0.69, 0.88) 0.035 | 0.81 (0.66, 0.91)  0.023 | 0.79 (0.63, 0.97) 0.01 |
| Q3 | 0.64 (0.54, 0.76) <0.0001 | 0.50  (0.43, 0.58) <0.0001 | 0.45 (0.39, 0.52) <0.0001 | 0.71 (0.60, 0.85) 0.0002 | 0.58 (0.49, 0.68) <0.0001 | 0.53 (0.46, 0.62) <0.0001 | 0.78 (0.66, 0.89) 0.033 | 0.74 (0.62, 0.91) <0.014 | 0.71 (0.60, 0.94) <0.0001 |
| Q4 | 0.34 (0.28, 0.42) <0.0001 | 0.27  (0.22,  0.32) <0.0001 | 0.24 (0.20, 0.28) <0.0001 | 0.42 (0.33, 0.52) <0.0001 | 0.34 (0.28, 0.41) <0.0001 | 0.31 (0.25, 0.37) <0.0001 | 0.77 (0.64, 1.07) 0.06 | 0.72  (0.72, 1.19)  0.12 | 0.89  (0.77, 1.21) 0.19 |
| P for trend | <0.0001 | <0.0001 | <0.0001 | <0.0001 | <0.0001 | <0.0001 | <0.0001 | <0.0001 | <0.0001 |
| Change per quartile | 0.70 (0.66, 0.75)  <0.0001 | 0.63  (0.60,  0.67)  <0.0001 | 0.60 (0.57, 0.64) <0.0001 | 0.75 (0.70, 0.81) <0.0001 | 0.69 (0.65,  0.73)  <0.0001 | 0.66 (0.63,  0.70)  <0.0001 | 0.79  (0.66, 0.84) 0.03 | 0.74  (0.61,  0.92)  0.01 | 0.71  (0.55,  0.98)  <0.0001 |

Model 1: adjust for none.

Model 2: adjust for gender; coronary; age; hypertension; diabetes; ARDS; COPD; renal failure.

Model3: adjust for heart rate; SBP; mean arterial pressure; diastolic pressure; respire rate; temperature; SPO2； platelet; potassium; sodium; creatinine; hemoglobin; WBC; AST, BNP, LVEF, diuretic, ACEI/ARB/ARNI, cardiac glycoside.

### Table S3. minimum albumin and the risk of all-cause mortality by Cox proportional hazards

|  | HR, 95%CI, P | | | | | | | | |
| --- | --- | --- | --- | --- | --- | --- | --- | --- | --- |
|  | Model 1 | | | Model 2 | | | Model 3 | | |
|  | 14th day | 28th day | 90th day | 14th day | 28th day | 90th day | 14th day | 28th day | 90th day |
| Continuous | 0.58 (0.53,  0.64)  <0.0001 | 0.49  (0.45,  0.54)  <0.0001 | 0.46 (0.42,  0.50)  <0.0001 | 0.64 (0.58,  0.71)  <0.0001 | 0.56 (0.51,  0.61)  <0.0001 | 0.52 (0.48,  0.57)  <0.0001 | 0.43  (0.31,  0.88)  <0.0001 | 0.33 (0.21,  0.91)  <0.0001 | 0.30 (0.19,  0.92)  <0.0001 |
| Categorical |  |  |  |  |  |  |  |  |  |
| Q1 | 1.0 | 1.0 | 1.0 | 1.0 | 1.0 | 1.0 | 1.0 | 1.0 | 1.0 |
| Q2 | 0.77 (0.65, 0.91) 0.0018 | 0.67  (0.58, 0.77) <0.0001 | 0.64 (0.56, 0.73) <0.0001 | 0.83 (0.70, 0.98) 0.0280 | 0.74 (0.64, 0.86) <0.0001 | 0.72 (0.63, 0.82) <0.0001 | 0.55 (0.44, 0.98) 0.014 | 0.51 (0.43, 0.98) <0.0001 | 0.44 (0.39, 0.98) <0.0001 |
| Q3 | 0.64 (0.54, 0.76) <0.0001 | 0.50 (0.43, 0.58) <0.0001 | 0.45 (0.39, 0.52) <0.0001 | 0.71 (0.60, 0.85) 0.0002 | 0.58 (0.49, 0.68) <0.0001 | 0.53 (0.46, 0.62) <0.0001 | 0.51 (0.33, 0.86) 0.0006 | 0.42 (0.30, 0.93) <0.0001 | 0.40 (0.28, 0.95) <0.0001 |
| Q4 | 0.34 (0.28, 0.42) <0.0001 | 0.27 (0.22, 0.32) <0.0001 | 0.24 (0.20, 0.28) <0.0001 | 0.42 (0.33, 0.52) <0.0001 | 0.34 (0.28, 0.41) <0.0001 | 0.31 (0.25, 0.37) <0.0001 | 0.49 (0.31, 0.87) <0.0001 | 0.41 (0.28, 0.89) <0.0001 | 0.39  (0.26, 0.91) <0.0001 |
| P for trend | <0.0001 | <0.0001 | <0.0001 | <0.0001 | <0.0001 | <0.0001 | <0.0001 | <0.0001 | <0.0001 |
| Change per quartile | 0.70 (0.66, 0.75)  <0.0001 | 0.63 (0.60,  0.67)  <0.0001 | 0.60 (0.57, 0.64) <0.0001 | 0.75 (0.70, 0.81) <0.0001 | 0.69 (0.65,  0.73)  <0.0001 | 0.66 (0.63,  0.70)  <0.0001 | 0.67  (0.45, 0.91) <0.0001 | 0.59  (0.41,  0.92)  <0.0001 | 0.50  (0.39,  0.93)  <0.0001 |

Model 1: adjust for none.

Model 2: adjust for gender; coronary; age; hypertension; diabetes; ARDS; COPD; renal failure.

Model3: adjust for heart rate; SBP; mean arterial pressure; diastolic pressure; respire rate; temperature; SPO2； platelet; potassium; sodium; creatinine; hemoglobin; WBC; AST, BNP, LVEF, diuretic, ACEI/ARB/ARNI, cardiac glycoside.

**Table S4. Mortality rates of patients with different albumin levels at 14th, 28th and 90th days after admission to intensive care unit**

| Parameters | 14 day-mortality | 28 day-mortality | 90 day-mortality |
| --- | --- | --- | --- |
| Q1(Albumin≤0.94mg/dl) | 17.9 | 26.3 | 30.7 |
| Q2(Albumin0.94-1.29mg/dl) | 13.9 | 18.7 | 20.3 |
| Q3(Albumin1.29-1.90mg/dl) | 11.7 | 13.7 | 14.6 |
| Q4(Albumin1.90-15.53mg/dl) | 6.5 | 7.6 | 7.9 |

**Table S5.** Threshold effect analysis of association between albumin (mg/dl) and all-cause mortality

|  | 14 day-mortality | | 28 day-mortality | | 90 day-mortality | |
| --- | --- | --- | --- | --- | --- | --- |
|  | β(95%CI) | P | β(95%CI) | P | β(95%CI) | P |
| All-cause mortality |  |  |  |  |  |  |
| Fitting by the standard linear model | 0.58 (0.53, 0.64) | <0.0001 | 0.49 (0.45, 0.54) | <0.0001 | 0.46 (0.42, 0.50) | <0.0001 |
| Fitting by the two-piecewise linear model |  |  |  |  |  |  |
| Inﬂection point | 3.56 |  | 3.56 |  | 3.5 |  |
| UA＜Threshold | 0.65 (0.56, 0.74) | <0.0001 | 0.52 (0.47, 0.59) | <0.0001 | 0.48 (0.43, 0.54) | <0.0001 |
| UA ≥ Threshold | 0.37 (0.25, 0.56) | <0.0001 | 0.37 (0.26, 0.53) | <0.0001 | 0.37 (0.27, 0.51) | <0.0001 |
| Log likelihood ratio | 0.021 |  | 0.107 |  | 0.158 |  |

**Table S6. Multiplicative statistical interaction of covariates**

|  | 14 day-mortality | | | 28 day-mortality | | | | 90 day-mortality | | | |
| --- | --- | --- | --- | --- | --- | --- | --- | --- | --- | --- | --- |
|  | β(95%CI) | P | P (interaction) | β(95%CI) | P | P (interaction) | β(95%CI) | | P | P  (interaction) | |
| Hypertensive |  |  | <0.0001 |  |  | <0.0001 |  | |  | <0.0001 | |
| 0 | 0.68(0.61-0.77) | <0.0001 |  | 0.58(0.52-0.64) | <0.0001 |  | 0.53(0.48-0.58) | | <0.0001 |  | |
| 1 | 0.44(0.37-0.52) | <0.0001 |  | 0.37(0.31-0.43) | <0.0001 |  | 0.35(0.30-0.41) | | <0.0001 |  | |
| Age |  |  | 0.003 |  |  | 0.003 |  | |  | 0.002 | |
| ＜years | 0.49(0.42-0.58) | <0.0001 |  | 0.43(0.37-0.68) | <0.0001 |  | 0.40(0.35-0.45) | | <0.0001 |  | |
| ≥years | 0.67(0.59-0.76) | <0.0001 |  | 0.56(0.50-0.62) | <0.0001 |  | 0.52(0.46-0.58) | | <0.0001 |  | |
| Coronary |  |  | 0.40 |  |  | 0.13 |  | |  | 0.22 | |
| 0 | 0.61(0.54-0.69) | <0.0001 |  | 0.53(0.48-0.59) | <0.0001 |  | 0.49(0.45-0.54) | | <0.0001 |  | |
| 1 | 0.56(0.47-0.66) | <0.0001 |  | 0.46(0.40-0.54) | <0.0001 |  | 0.44(0.38-0.51) | | <0.0001 |  | |
| Diabetes |  |  | 0.71 |  |  | 0.54 |  | |  | 0.93 | |
| 0 | 0.59(0.52-0.66) | <0.0001 |  | 0.50(0.45-0.56) | <0.0001 |  | 0.46(0.42-0.51) | | <0.0001 |  | |
| 1 | 0.57(0.48-0.67) | <0.0001 |  | 0.48(0.41-0.55) | <0.0001 |  | 0.46(0.40-0.53) | | <0.0001 |  | |
| COPD |  |  | 0.35 |  |  | 0.001 |  | |  | 0.35 | |
| 0 | 0.57(0.52-0.63) | <0.0001 |  | 0.45(0.40-0.51) | <0.0001 |  | 0.45(0.42-0.49) | | <0.0001 |  | |
| 1 | 0.73(0.45-1.17) | 0.19 |  | 0.60(0.54-0.68) | <0.0001 |  | 0.55(0.37-0.83) | | 0.004 |  | |
| Renal failure |  |  | 0.14 |  |  | 0.001 |  | |  | 0.001 | |
| 0 | 0.54(0.47-0.63) | <0.0001 |  | 0.49(0.45-0.53) | <0.0001 |  | 0.42(0.37-0.48) | | <0.0001 | |  |
| 1 | 0.70(0.61-0.80) | <0.0001 |  | 0.58(0.38-0.88) | <0.0001 |  | 0.56(0.50-0.62) | | <0.0001 | |  |
| ARDS |  |  | 0.0003 |  |  | 0.009 |  | |  | | 0.04 |
| 0 | 0.55(0.50-0.61) | <0.0001 |  | 0.48(0.44-0.52) | <0.0001 |  | 0.45(0.41-0.49) | | <0.0001 | |  |
| 1 | 1.06(0.76-1.50) | 0.72 |  | 0.71(0.54-0.94) | 0.02 |  | 0.60(0.46-0.77) | | <0.0001 | |  |
| Cigarette |  |  | 0.92 |  |  | 0.51 |  | |  | | 0.56 |
| 1 | 0.51(0.34-0.75) | 0.0008 |  | 0.48(0.34-0.68) | 0.97 |  | 0.46(0.33-0.63) | | <0.0001 | |  |
| 2 | 0.46(0.22-0.97) | 0.04 |  | 0.53(0.27-1.03) | 0.06 |  | 0.52(0.27-1.00) | | 0.05 | |  |
| 3 | 4.66(0.75-28.81) | 0.09 |  | 1.16(0.27-5.00) | 0.84 |  | 1.16(0.27-5.02) | | 0.85 | |  |
| 4 | 0.52(0.36-0.75) | 0.0005 |  | 0.41(0.30-0.57) | <0.0001 |  | 0.42(0.30-0.57) | | <0.0001 | |  |
| Gender |  |  | 0.51 |  |  | 0.43 |  | |  | | 0.47 |
| Men | 0.60(0.52-0.68) | <0.0001 |  | 0.51(0.45-0.57) | <0.0001 |  | 0.47(0.42-0.52) | | <0.0001 | |  |
| Women | 0.56(0.48-0.65) | <0.0001 |  | 0.47(0.42-0.54) | <0.0001 |  | 0.44(0.39-0.50) | | <0.0001 | |  |

**Table S7. Albumin and the risk of all-cause mortality by Cox proportional hazards**

|  | HR, 95%CI, P | | | | | | | | |
| --- | --- | --- | --- | --- | --- | --- | --- | --- | --- |
|  | Model 1 | | | Model 2 | | | Model 3 | | |
| ARDS | 14^th^ day | 28^th^ day | 90^th^ day | 14^th^ day | 28^th^ day | 90^th^ day | 14^th^ day | 28^th^ day | 90^th^ day |
| 0 | 0.71 (0.67, 0.76)  <0.0001 | 0.48 (0.44,  0.53)  <0.0001 | 0.45 (0.41,  0.49)  <0.0001 | 0.76 (0.71,  0.81)  <0.0001 | 0.54 (0.49,  0.59)  <0.0001 | 0.51 (0.47,  0.56)  <0.0001 | 0.77 (0.71,  0.83)  <0.0001 | 0.55 (0.49,  0.62)  <0.0001 | 0.52 (0.46,  0.57)  <0.0001 |
| 1 | 1.05 (0.85, 1.30) 0.6747 | 0.70 (0.53, 0.93) 0.0139 | 0.58 (0.45, 0.76) <0.0001 | 1.07 (0.86, 1.32) 0.5525 | 0.76 (0.57, 1.01) 0.0555 | 0.64 (0.49, 0.83) 0.0008 | 1.04 (0.80, 1.35) 0.7858 | 0.77 (0.55, 1.08) 0.1344 | 0.64 (0.47, 0.87) 0.0047 |

Model 1: adjust for none.

Model 2: adjust for gender, coronary, age, hypertension, diabetes, ARDS, COPD, renal failure.

Model3: adjust for heart rate, SBP, mean arterial pressure, diastolic pressure, respire rate, temperature, SPO2, platelet, potassium, sodium, creatinine, hemoglobin, WBC, AST.
